# Low-Light Image Segmentation on Edge Computing System

**DOI:** 10.3390/s26010327

**Published:** 2026-01-04

**Authors:** Sung-Chan Choi, Sung-Yeon Kim

**Affiliations:** 1 Department of Electrical and Electronic Engineering, Yonsei University, Seoul 03722, Republic of Korea; 2 Autonomous IoT Research Center, Korea Electronics Technology Institute, Seongnam 13509, Republic of Korea; csc@keti.re.kr; 3 Department of IT Convergence, ICT Polytech Institute of Korea, Gwangju 12777, Republic of Korea

**Keywords:** low-light image, image segmentation, deep learning, edge computing

## Abstract

Segmenting low-light images, such as images showing cracks on tunnel walls, is challenging due to limited visibility. Hence, we need to combine image brightness enhancement and a segmentation algorithm. We introduce essential preliminaries, specifically highlighting deep learning-based low-light image enhancement methods and the pixel-level image segmentation algorithm. After that, we provide a three-step low-light image segmentation algorithm. The proposed algorithm begins with brightness and contrast enhancement of low-light images, followed by accurate segmentation using a U-Net model. By various experimental results, we show the performance metrics of the proposed low-light image segmentation algorithm and compare the proposed algorithm’s performance against several baseline models. Furthermore, we demonstrate the implementation of the proposed low-light image segmentation pipeline on an edge computing platform. The implementation results show that the proposed algorithm is sufficiently fast for real-time processing.

## 1. Introduction

The concrete structures are foundational to modern infrastructure which forms the backbone of buildings, roads, bridges, and various civil engineering facilities. However, the concrete structures may have cracks due to various reasons, e.g., environmental conditions, mechanical stresses, and material degradations, and these cracks not only compromise the structural integrity but also pose significant safety risks to users. For example, undetected cracks have led to severe consequences, including economic losses and public safety hazards. Hence, the early detection and monitoring of concrete cracks have emerged as a crucial research area [1,2,3].

Traditional methods for monitoring concrete cracks predominantly rely on manual visual inspections conducted by trained personnel. This manual monitoring method is widely adopted in the real-world field, but this method may be inherently limited by human subjectivity, variability in judgment, and inefficiencies in large-scale or complex environments. Additionally, it is difficult to provide continuous, quantitative data for long-term monitoring in the manual inspections.

To address these challenges in crack monitoring, there have been many works using machine learning techniques in [4,5,6,7,8,9,10,11,12,13,14,15,16,17]. The convolutional neural network (CNN) which is specialized in image recognition can be used to detect crack damages, and its usages are proposed in [4,5,6]. These studies process high-resolution images by using the CNN algorithm to achieve fast and accurate detection rates for real-time inspections. To increase the crack detection efficiency, enhanced CNN algorithms are used, e.g., combining them with Naïve Bayes data fusion, in [7,8,9,10,11]. In other ways, CNN algorithms integrate with other techniques, e.g., simultaneous data acquisition, Stockwell transformation, or 3D shadow modeling, to detect crack damages in [12,13,14,15,16,17].

Despite the above advancement, the studies have some limitations related to the tunnel crack detection characteristics. First, most tunnel crack images are low-resolution, i.e., tunnel crack images frequently suffer from non-uniform or insufficient illumination in real inspection environments. Although artificial lighting is typically installed on inspection vehicles, the large structural scale of tunnels, limited mounting positions for lighting modules, and the highly absorptive concrete surface often lead to severe shadowed regions and local dark areas. As a result, cracks commonly appear in low-light or unevenly illuminated regions. Hence, the method should consider crack detection in low-resolution images. Second, tunnel crack detection should be operated in real-time while the crack detector, e.g., mobile vehicle, moves through the tunnel. Hence, the algorithms should be sufficiently light to operate in mobile vehicles which have low computational processing power.

Various studies have been performed to achieve crack damage detection in low-light images [18,19,20,21,22,23,24,25,26,27,28,29,30,31,32]. In [18,19,20,21], a hybrid crack detection algorithm is proposed that combines histogram-based thresholding with mathematical morphology to achieve enhanced crack segmentation accuracy. To improve recognition efficiency, a pixel-level segmentation algorithm is used in [22,23,24]. There are several studies that apply deep-learning-based segmentation architectures such as U-Net, SegNet, and ResNet for crack detection in low-light environments [25,26,27,28,29,30,31,32]. However, these architectures typically contain tens of millions of parameters and require tens to hundreds of GFLOPs per inference; for example, a baseline U-Net has approximately 31 M parameters, SegNet exceeds 29 M parameters, and ResNet-based decoders often require over 10–15 GFLOPs. Such computational demands make these methods impractical for real-time deployment on mobile vehicles or edge computing devices with limited processing resources.

The above studies propose various techniques to detect crack damages in low-light images; however, these studies propose algorithms designed for environments with generally high computing power. As we mentioned earlier, tunnel crack detection often requires real-time operation on mobile vehicles, i.e., immediate on-site responses are necessary. Hence, tunnel crack detection techniques need to be operated within a lightweight computing environment which can be installed on the edge computing device or even on the mobile vehicles.

In this paper, we propose an integrated approach for low-light image segmentation operating with an edge computing platform which has generally low computing power. Specifically, we develop an advanced image enhancement algorithm to improve the image visibility caused by the non-uniform lighting conditions. While previous works focused primarily on image enhancement or segmentation individually, our method introduces a tightly coupled enhancement–segmentation pipeline optimized for real-time edge computing. The proposed algorithm is designed to overcome the computational constraints, i.e., low computing power, of edge computing or mobile vehicle environments. To enable efficient deployment on edge devices, our approach does not modify the U-Net architecture itself; instead, computational optimization is achieved through the pre-processing pipeline. By applying Enlighten-GAN-based brightness enhancement followed by contrast adjustment, the input images are normalized and made more informative before entering U-Net. This reduces the feature extraction burden on the segmentation network and significantly shortens the overall inference time, allowing the complete pipeline to operate reliably on low-power edge computing platforms. We can summarize the contributions of this paper as follows: First, we design an illumination–contrast enhancement pipeline that combines GAN-based illumination correction with lightweight contrast normalization to improve the visibility of crack structures under non-uniform lighting conditions. Second, we develop a deep learning-based low-light image segmentation algorithm optimized for edge devices, ensuring practical deployment in real-world conditions. Last, we verify the proposed algorithm through extensive experiments, demonstrating its robustness and accuracy in various low-light and complex environmental scenarios.

Since the goal of this work is to design an illumination–contrast enhancement pipeline that enables reliable crack segmentation under strict edge computing constraints, we intentionally adopt the standard U-Net as a fixed segmentation backbone rather than comparing alternative segmentation architectures. This allows the contribution of the enhancement stages to be isolated and evaluated without introducing variations in computational cost or model complexity, which would violate the constraints of the target embedded platform. Note that the scientific contribution of this work does not lie in proposing a new network architecture, but in analyzing how different illumination enhancement mechanisms interact with pixel-level crack segmentation under real low-light tunnel conditions. Prior studies have treated enhancement and segmentation as independent modules, while this work provides a systematic investigation of their coupling behavior and its implications for edge computing deployment. This analysis-oriented perspective distinguishes the proposed study from a simple combination of existing techniques.

This paper is organized as follows: In Section 2, we review the background and related works of image enhancement algorithms and the pixel-level segmentation algorithm. In Section 3, we propose the low-light image segmentation algorithm, and in Section 4, we provide the experimental results with the performance and practical applicability of the proposed approach. Finally, Section 5 concludes with a summary of the proposed algorithm.

## 2. Background and Related Work

This section reviews classical and deep learning-based techniques relevant to non-uniform illumination enhancement and crack segmentation, providing the technical background that motivates the design of the proposed pipeline. We first summarize representative non-ML approaches for handling low-light and uneven illumination conditions, followed by modern deep learning enhancement models and the U-Net segmentation background used in our method.

### 2.1. Illumination Enhancement Method

#### 2.1.1. Non-ML-Based Method

Prior to the emergence of deep learning, numerous classical image enhancement methods were proposed to correct illumination imbalance and improve visibility in degraded scenes. Nonlinear diffusion filtering [33] enhances contrast by suppressing noise through anisotropic diffusion, but its local gradient formulation limits performance under strong non-uniform lighting. Retinex-based models such as the plug-and-play Retinex framework [34] provide strong illumination estimation yet rely on iterative optimization, making them unsuitable for real-time edge deployment. Physics-based enhancement approaches [35] utilize illumination transport models and produce globally consistent brightness correction, but may oversmooth thin crack structures. Recent biologically inspired decomposition methods [36] and multi-layer lightness–statistics techniques [37] preserve the natural appearance of the scene but involve multiple computational stages and lack robustness in highly shadowed tunnel environments. These classical approaches form the foundation of illumination correction research; however, their computational complexity and limited adaptability to severe lighting variation motivate the use of lightweight deep enhancement models for edge-based crack inspection.

#### 2.1.2. ML-Based Method

The Zero-DCE [38] is a lightweight deep model that enhances illumination by predicting pixel-wise curve parameters without requiring paired training data. The estimated curves are iteratively applied to correct global and local lighting imbalance. With around 79 K parameters and approximately 1.77 GFLOPs per 256 × 256 image, Zero-DCE is highly suitable for edge deployment. In this study, Zero-DCE is considered as a candidate enhancement model to assess the influence of illumination correction on crack segmentation accuracy. The Enlighten-GAN [39] performs unpaired low-light enhancement using a GAN framework with global–local discriminators. Its ability to learn illumination statistics without paired data makes it robust in real-world scenarios where controlled lighting is difficult to achieve. Enlighten-GAN preserves structural details and provides strong correction of non-uniform illumination in a single forward pass, which is advantageous for real-time edge computing. For this reason, Enlighten-GAN is adopted as the primary illumination correction module in our pipeline. Although Enlighten-GAN is heavier than Zero-DCE, it remains practical for edge inference due to its single-pass generator architecture consisting of approximately 8M parameters, which is significantly smaller than typical GAN-based enhancement models. The SID model [40] enhances raw Bayer images captured in extreme low-light conditions by mapping them directly to sRGB space. Although SID achieves strong illumination recovery, its requirement for raw sensor input and its high computational complexity make it less suitable for embedded inspection platforms. The SID requires substantially higher computational cost due to its raw-domain processing and deep encoder–decoder structure, making it less suitable for edge devices.

### 2.2. Segmentation Method

The U-Net framework [41] is a widely used encoder–decoder architecture for pixel-level segmentation. Its skip connections preserve fine-grained spatial information, making it effective for detecting thin crack structures. Due to its relatively small computational footprint compared with modern transformer-based segmentation models, U-Net is adopted as a fixed and lightweight backbone in this study. This allows the contribution of the enhancement modules to be isolated, enabling a fair evaluation of illumination–contrast pre-processing under edge computing constraints. Beyond U-Net, recent segmentation studies such as masked-supervised learning approaches [42] have explored label-efficient and structure-aware supervision to improve segmentation accuracy. While such architectures provide valuable advances for high-resource environments, the goal of this work is not to introduce a new segmentation backbone but to evaluate how illumination enhancement influences segmentation performance under edge computing constraints.

## 3. Low-Light Image Segmentation Algorithm

The goal of this section is to provide a clear and reproducible description of the entire pipeline, including the pre-processing operations, model configuration, training procedure, and inference process. In this section, we propose a low-light image segmentation algorithm which is designed as a three-step process. In the first step, the brightness of low-light images is enhanced by using the deep learning-based image enhancement algorithm, i.e., Enlighten-GAN algorithm, which has a unique strength in handling illumination and noise of images. As we will mention in the next section, the Enlighten-GAN model shows the best performance to brighten low-light images among the candidate models. In the next step, the contrast of the brightness-enhanced image is optimized. This step enhances visibility and feature clarity, providing a better input for the subsequent segmentation step. In the last step, the enhanced low-light image is used as the input to the pixel-level segmentation algorithm. In this work, the U-Net architecture itself is unchanged. Instead, we define a modified U-Net pipeline as the segmentation pipeline that applies Enlighten-GAN-based brightness enhancement and contrast optimization prior to applying the standard U-Net. This pre-processing modification significantly improves segmentation performance under low-light conditions without altering U-Net’s internal structure. The detailed procedures are described in the following subsections.

### 3.1. Illumination Enhancement

The first stage applies Enlighten-GAN to improve global and local illumination of low-light tunnel images. Enlighten-GAN operates in a single-pass configuration using the pretrained generator for computational efficiency. Given a low-light RGB image $I_{raw}$, the generator produces the illumination-corrected output:

(1) $$I_{bright}\leftarrow f_{\mathrm{brightness}}\left(I_{raw}\right),$$

where $f_{\mathrm{brightness}}$ denotes the brightness enhancement function. Before enhancement, each input is resized to 256×256 and normalized to the range [0,1]. Although Enlighten-GAN introduces approximately 11 GFLOPs per image, its illumination correction substantially enhances structural visibility in low-light images. This improvement reduces the burden on the downstream U-Net segmentation by exposing fine crack boundaries that would otherwise remain obscured. Note that the goal of the enhancement stage is not to modify the Enlighten-GAN architecture itself, but to construct an illumination–contrast pipeline that produces edge-preserving inputs suitable for crack segmentation. Classical global operators such as gamma correction tend to amplify brightness uniformly and often fail to recover crack boundaries in shadowed regions, while Enlighten-GAN provides locally adaptive illumination correction in a single forward pass, which is advantageous for edge deployment.

### 3.2. Contrast Enhancement

While Enlighten-GAN corrects illumination, its output often exhibits limited contrast near fine edges. To reinforce structural separability, a lightweight contrast normalization operator is applied:

(2) $$I_{const}\leftarrow f_{\mathrm{contrast}}\left(I_{bright}\right),$$

where $f_{\mathrm{contrast}}$ is a contrast normalization function. This operation expands the intensity range of pixels relative to their surroundings, making the boundaries more distinguishable for U-Net. In particular, regions that remain ambiguous after the Enlighten-GAN enhancement, e.g., cracks embedded in weak shadows or shallow illumination fall-off, become more separable due to the increased gradient response. This step is computationally negligible and does not introduce artifacts, but significantly improves the downstream segmentation accuracy, making it suitable for edge devices.

### 3.3. Image Segmentation

The final stage performs pixel-level segmentation using the standard U-Net architecture. The contrast-enhanced image $I_{const}$ is resized to 256×256 and normalized before being fed into U-Net, and the segmented result is made by using the U-Net as follows:

(3) $$C_{segment}\leftarrow f_{\mathrm{segment}}\left(I_{const}\right),$$

where $C_{segment}$ denotes the resulting segmentation mask. To ensure reproducibility, the training configuration is described as follows. The dataset used for segmentation is split into 70% for training, 15% for validation, and 15% for testing. Data augmentation consists of random rotations, horizontal flipping, and brightness jittering. The network is trained for 200 epochs using the Adam optimizer and Dice Loss is employed to mitigate the strong imbalance between crack and non-crack pixels. Although the baseline U-Net contains approximately 31 million parameters and requires 60–80 GFLOPs for a 256×256 input, the preceding enhancement stages simplify its feature extraction, leading to stable inference performance on edge computing platforms without modifying the U-Net architecture. During inference, a fixed threshold of 0.5 is applied to obtain the binary mask, and to suppress small isolated false positives, we apply a morphological opening operation with a 3×3 structuring element as a post-processing step. This refinement improves spatial consistency in the predicted crack regions and enhances the robustness of the segmentation results on edge devices. All the above algorithm steps are summarized in Algorithm 1.
**Algorithm 1** Low-light Image Segmentation Algorithm1:**Input**: Original image of tunnel crack $I_{raw}$2:**Step 1**: Get the brightness enhancement image $I_{bright}$ by using Enlighten-GAN algorithm.3:$I_{bright}\leftarrow f_{\mathrm{brightness}}\left(I_{raw}\right)$4:**Step 2**: Get the contrast enhancement image $I_{const}$ by using the contrast enhancement algorithm.5:$I_{const}\leftarrow f_{\mathrm{contrast}}\left(I_{bright}\right)$6:**Step 3**: Get the segmentation result by using the U-Net algorithm.7:$C_{segment}\leftarrow f_{\mathrm{segment}}\left(I_{const}\right)$8:**Return** $C_{segment}$

## 4. Experimental Result

In this section, we provide some experimental results of the proposed low-light image segmentation algorithm. We first show the image refinement results by image enhancement models, i.e., zero-DCE, Enlighten-GAN, and SID models. We compare the performance of image enhancement models to apply the low-light image segmentation model, i.e., U-Net learning model, and provide the performance of the proposed low-light image segmentation algorithm. We evaluate segmentation performance using widely adopted pixel-level metrics, including Precision, Recall, Dice Score, Dice Loss, and IoU, following their standard definitions in the image segmentation literature (see Appendix A for details).

### 4.1. Experimental Setup

All experiments are conducted using both a desktop workstation and an edge computing platform. The desktop system consists of an Intel Core i9-10900K CPU, 64 GB RAM, and an NVIDIA RTX 3090 GPU, which are used for model training. The edge device used for deployment evaluation is the NVIDIA Jetson Xavier AGX/NX Jetpack 4.5.1. The crack dataset consists of a total of 580 annotated tunnel images. We randomly split the dataset into 70% for training, 15% for validation, and 15% for testing. All images are resized to 256×256 during training and inference to ensure consistency with the edge-device configuration.

### 4.2. Image Enhancement Performance

In this subsection, we show the image refinement results by applying the image enhancement models, and we analyze the enhanced images by using the image histogram method, which is a graphical representation that illustrates the distribution of pixel intensity values, providing insight into its tonal and color characteristics. The image histogram consists of two primary axes: the x-axis, which represents the range of pixel intensity values, e.g., 0 to 255 for 8-bit images, where 0 corresponds to black and 255 to white in grayscale, and the y-axis, which indicates the frequency or number of pixels at each intensity value. For example, if most pixels are concentrated on the left, then the image is underexposed, if most pixels are concentrated on the right, then the image is overexposed, and if the distribution is even across the range, then the image is well-balanced.

**Figure 1:**
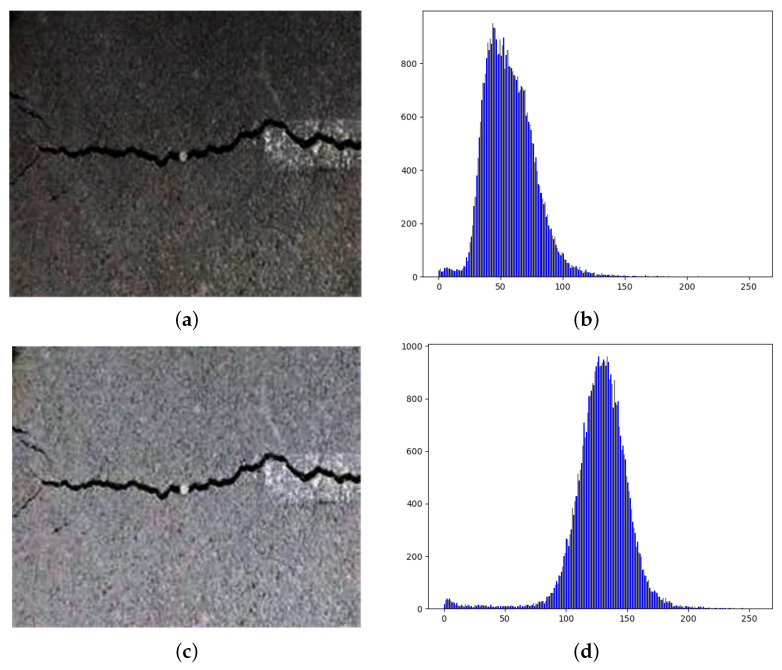

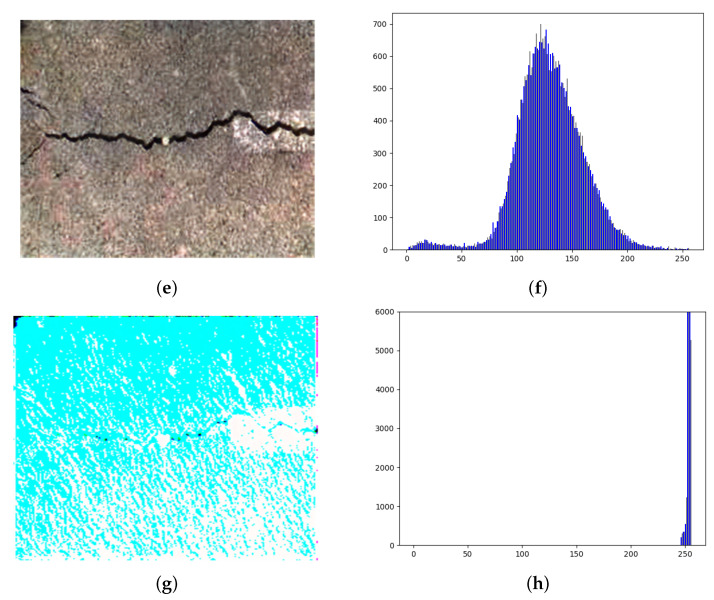
Tunnel crack image and its histogram distribution. (**a**) Low-light tunnel crack image; (**b**) low-light tunnel crack image histogram; (**c**) zero-DCE-enhanced tunnel crack image; (**d**) zero-DCE-enhanced tunnel crack image histogram; (**e**) Enlighten-GAN-enhanced tunnel crack image; (**f**) Enlighten-GAN-enhanced tunnel crack image histogram; (**g**) SID-enhanced tunnel crack image; (**h**) SID-enhanced tunnel crack image histogram.

In Figure 1, we show both the low-light image and enhanced images with the corresponding histogram distribution. The histogram distribution of the low-light image shows a dark pixel distribution, which indicates the low-illumination conditions of tunnels. The image brightness is enhanced using deep learning-based image enhancement algorithms, i.e., Zero-DCE, Enlighten-GAN, and SID, as we already mentioned before. In zero-DCE enhancement, the histogram distribution shows a significant reduction in the dark pixel regions, and we can find that the image’s overall illumination is improved. Similarly, the enhanced image applied by Enlighten-GAN shows a reduction in dark pixel distribution and an enhancement of the image’s brightness. However, in SID mode, we can find that only the very bright pixels remain, i.e., the image’s brightness is overexposed, while the dark pixel distribution is completely reduced. This indicates that the SID model may produce a loss of image detail.

### 4.3. Accuracy Performance

**Figure 2:**
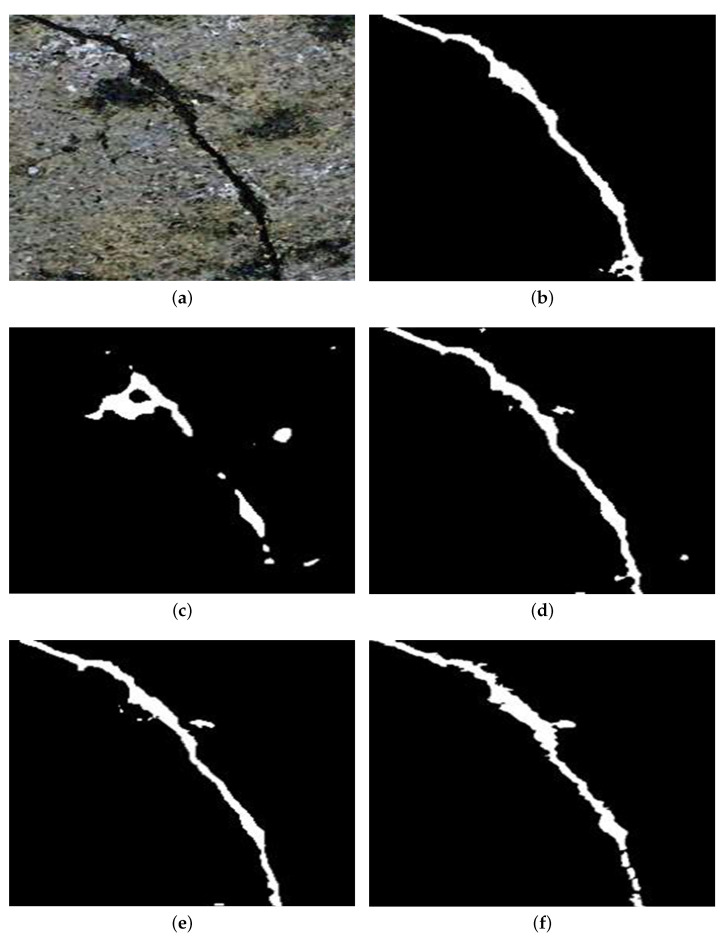
The low-light image segmentation results. (**a**) Low-light tunnel crack image; (**b**) segmentation result without image enhancement; (**c**) segmentation result with SID enhancement; (**d**) segmentation result with zero-DCE enhancement; (**e**) segmentation result with enlighten-GAN enhancement; (**f**) segmentation result with enlighten-GAN+CE enhancement.

In Figure 2, we provide the low-light image segmentation results. We show that the only SID-enhanced image produces an unexpected result, while the other images, i.e., low-light image, zero-DCE-enhanced image, enlighten-GAN-enhanced image, and enlighten-GAN+CE-enhanced image, produce suitable results to detect a tunnel crack. As mentioned earlier, this result can be expected because the SID model may produce an overexposure phenomenon during the image enhancement and this has a negative impact on low-light image segmentation. Furthermore, note that our proposed low-light image segmentation algorithm, i.e., Figure 2f, provides a clearer result than other algorithms.

### 4.4. Performance Comparison

**Figure 3:**
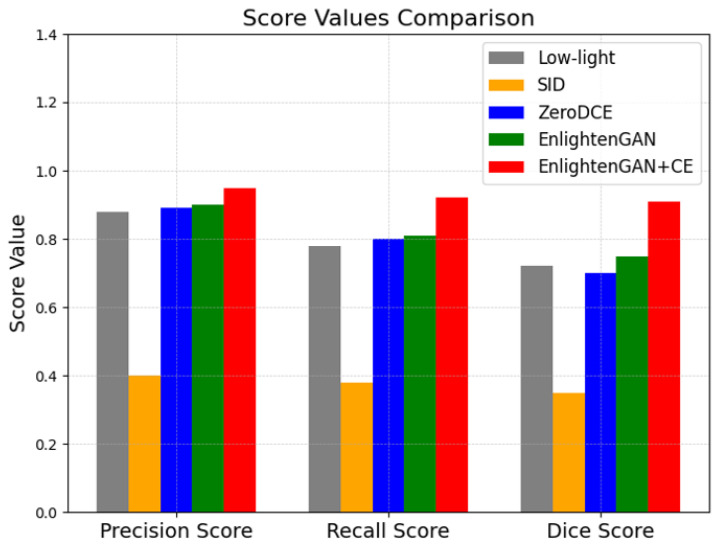
Accuracy performance of low-light image segmentation algorithms.

We compare the performance metrics of the low-light image segmentation algorithms. We use Precision Score, Recall Score, and Dice Score as the performance metrics. In Figure 3, we show the performance metrics of the candidate low-light image segmentation algorithms, i.e., SID, zeroDCE, and EnlightenGAN, and the proposed low-light image segmentation algorithm, i.e., EnlightenGAN+CE. The experimental results show that if the SID algorithm is used to improve the brightness of images, the accuracy performance severely decreases. This is an easily expected result because the SID model may produce an overexposure phenomenon during the image enhancement as mentioned earlier. On the other hand, the zero-DCE and Enlighten-GAN models show improved performance metrics compared to the SID model, but it is difficult to say that we can achieve clear enhancement compared to the original low-light image. However, the proposed low-light image segmentation algorithm, i.e., brightness and contrast enhancement with the U-Net algorithm, improves all the performance metrics compared to the low-light image and the other candidate low-light image segmentation algorithms.

Because the scope of this work is the design of an edge-feasible enhancement segmentation pipeline, we restrict our comparison to combinations of different enhancement methods rather than including computationally intensive state-of-the-art segmentation architectures that fall outside the constraints of edge deployment.

### 4.5. Implementation Consideration

In the practical applications, the low-light image segmentation can be performed by drones or robots, which are compact and have low-computing power. Hence, we evaluate the proposed low-light image segmentation algorithm on the Jetson Xavier AGX and NX devices, which are light AI computing platforms developed by NVIDIA. We measure the processing time between the low-light tunnel crack image input and the image segmentation result transmitted to the data server. When 50 tunnel crack images are input to the low-light image segmentation algorithm on the implementation device, the processing time is 8.88 s for the Jetson Xavier AGX and 10.69 s for the Jetson Xavier NX, i.e., a processing rate of 5.63 images per second for the Jetson Xavier AGX and 4.67 images per second for the Jetson Xavier NX. These results show that the proposed low-light image segmentation algorithm is sufficient to apply with the compact AI computing platform in real-world environments.

We further analyzed the computational cost on the Jetson Xavier platforms. The full enhancement–segmentation pipeline consumes approximately 1.2 GB of GPU memory on Jetson Xavier AGX and 1.0 GB on Xavier NX when processing images of size 256 × 256. The inference time for each module is as follows: Zero-DCE (2.1 ms), Enlighten-GAN (6.7 ms), contrast enhancement (1.2 ms), and the modified U-Net (8.3 ms), resulting in an end-to-end latency of 18.3 ms per image. The model sizes are 0.3 MB (Zero-DCE), 45 MB (Enlighten-GAN), and 32 MB (modified U-Net). These results confirm that the proposed approach is deployable within the computational and memory constraints of embedded edge AI devices.

## 5. Conclusions

In this paper, we provide a low-light image segmentation algorithm by integrating a deep learning-based image brightness enhancement method and pixel-level segmentation. The proposed low-light image segmentation consists of three-step algorithms which significantly improve the visibility of low-light images. Based on the various experimental results, we show that our approach outperforms several baseline models in terms of accuracy and robustness. Moreover, the real-world implementation on the edge computing platform confirmed that the proposed algorithm achieves sufficiently fast inference speed; hence, our approach is suitable for real-world image segmentation tasks. Furthermore, beyond the empirical accuracy improvements, this study provides scientific observations on how low-light enhancement modifies crack-boundary distributions, how contrast normalization compensates for residual illumination imbalance, and how these factors affect segmentation reliability under edge computing constraints. However, despite our contributions, the proposed method has several limitations. First, under extreme degradations, e.g., heavy sensor noise, strong motion blur, or severely attenuated crack boundaries, the proposed pipeline may fail, since the illumination correction alone cannot recover sufficient structural information for the U-Net to produce reliable segmentation. Second, very high-resolution images may require additional memory optimization on edge devices. Finally, if the enhancement models are trained on illumination statistics similar to our dataset, the performance may degrade when applied to tunnels with different surface materials or lighting setups. These weakness represent inherent limitations of enhancement–segmentation pipelines and motivate future research on noise-robust and blur-aware crack detection.

## Data Availability

Data are contained within the article.

## Appendix A

We define some performance metrics to evaluate the low-light image segmentation algorithm. We first define the predicted pixel value $p_{i}$ and the ground-truth pixel value $g_{i}$ as follows:

(A1) $$p_{i}=\left\{\begin{array}{cc} 1, & \mathrm{if}\;\mathrm{pixel}\;i\;\mathrm{is}\;\mathrm{predicted}\;\mathrm{as}\;a\;\mathrm{part}\;\mathrm{of}\;\mathrm{crack}\\ 0, & \mathrm{if}\;\mathrm{pixel}\;i\;\mathrm{is}\;\mathrm{predicted}\;\mathrm{as}\;a\;\mathrm{background}, \end{array}\right.$$

and

(A2) $$g_{i}=\left\{\begin{array}{cc} 1, & \mathrm{if}\;\mathrm{pixel}\;i\;\mathrm{is}\;a\;\mathrm{part}\;\mathrm{of}\;\mathrm{crack}\\ 0, & \mathrm{if}\;\mathrm{pixel}\;i\;\mathrm{is}\;a\;\mathrm{background}. \end{array}\right.$$

We have four cases of the pixel prediction. The true positive (TP) is defined as the case where the pixel was a part of the crack and is predicted as a part of the crack; then, we can define the number of TP case pixels as follows:

(A3) $$TP=\sum_{i=1}^{N}p_{i}g_{i},$$

where *N* is the total number of pixels. The second case is the false positive (FP) where the pixel was a background but is predicted as a part of crack, then we can define the number of FP case pixels as follows

(A4) $$FP=\sum_{i=1}^{N}p_{i}\left(1-g_{i}\right).$$

The third case is the false negative (FN) where the pixel was a part of the crack but is predicted as a background; then we can define the number of FN pixels as follows:

(A5) $$FN=\sum_{i=1}^{N}\left(1-p_{i}\right)g_{i}.$$

The last case, true negative (TN), is defined as follows: the pixel was a background and is predicted as a background; then we can define the number of TN pixels as follows:

(A6) $$TN=\sum_{i=1}^{N}\left(1-p_{i}\right)\left(1-g_{i}\right).$$

We now introduce some performance metrics, precision score, recall score, F1 score, dice score, dice loss, and IoU score, which are key evaluation metrics used to measure the performance of image segmentation models. These metrics provide insights into the model’s ability to predict target regions accurately while minimizing errors. The precision score is defined as the proportion of predicted positive pixels that are correctly classified as positive as

(A7) $$\mathrm{Precision}\;\mathrm{Score}=\frac{TP}{TP+FP}.$$

It reflects the model’s ability to avoid false positive predictions and is particularly useful in scenarios where false positives are costly.

The recall score, also known as Sensitivity, is the proportion of actual positive pixels that are correctly predicted as

(A8) $$\mathrm{Recall}\;\mathrm{Score}=\frac{TP}{TP+FN}.$$

It evaluates the model’s ability to capture all relevant positive pixels, reducing false negative predictions.

The dice score is the harmonic mean of precision score and recall score, providing a single metric to balance the trade-off between false positives and false negatives, which is defined as

(A9) $$\mathrm{Dice}\;\mathrm{Score}=\frac{2TP}{2TP+FP+FN}.$$

It is especially valuable in datasets with imbalanced classes, where both Precision and Recall are important.

The dice loss is a loss function derived from the dice score and is used to train segmentation models, and it is defined as

(A10) $$\mathrm{Dice}\;\mathrm{Loss}=1-\frac{2TP}{2TP+FP+FN}.$$

It quantifies the dissimilarity between the predicted mask and the ground-truth mask. A dice loss value closer to zero indicates a high similarity between the two masks, suggesting good segmentation performance.

The intersection over union (IoU) score measures the ratio of the overlap between the predicted and ground-truth masks to their combined area, defined as

(A11) $$\mathrm{IoU}\;\mathrm{Score}=\frac{TP}{TP+FP+FN}.$$

It is a widely used metric in segmentation tasks to evaluate the spatial alignment between predictions and ground truth. A higher IoU score indicates better alignment and segmentation accuracy.

## References

[1] Yao Y., Tung S.T.E., Glisic B. (2014). Crack Detection and Characterization Techniques—An Overview. Struct. Control Health Monit.

[2] Munawar H.S., Hammad A.W., Haddad A., Soares C.A.P., Waller S.T. (2021). Image-based Crack Detection Methods: A Review. Infrastructures.

[3] Kulkarni S., Singh S., Balakrishnan D., Sharma S., Devunuri S., Korlapati S.C.R. (2022). CrackSeg9k: A Collection and Benchmark for Crack Segmentation Datasets and Frameworks. ECCV.

[4] Zhang L., Yang F., Zhang Y.D., Zhu Y.J. Road Crack Detection Using Deep Convolutional Neural Network. Proceedings of the 2016 IEEE International Conference on Image Processing (ICIP).

[5] Cha Y.J., Choi W., Büyüköztürk O. (2017). Deep Learning-Based Crack Damage Detection Using Convolutional Neural Networks. Comput.-Aided Civ. Infrastruct. Eng..

[6] Dorafshan S., Thomas R.J., Maguire M. (2018). Comparison of Deep Convolutional Neural Networks and Edge Detectors for Image-Based Crack Detection in Concrete. Constr. Build. Mater..

[7] Chen F.C., Jahanshahi M.R. (2017). NB-CNN: Deep Learning-Based Crack Detection Using Convolutional Neural Network and Naïve Bayes Data Fusion. IEEE Trans. Ind. Electron..

[8] Zou Q., Zhang Z., Li Q., Qi X., Wang Q., Wang S. (2018). DeepCrack: Learning Hierarchical Convolutional Features for Crack Detection. IEEE Trans. Image Process..

[9] Han J.H., Cho Y.C., Lee H.G., Yang H.S. Crack Detection Method on Surface of Tunnel Lining. Proceedings of the 2019 34th International Technical Conference on Circuits/Systems, Computers and Communications (ITC-CSCC).

[10] Gong Q., Wang Y., Yu Z., Zhu L., Shi H. A Tunnel Crack Identification Algorithm with Convolutional Neural Networks. Proceedings of the 2018 IEEE 4th Information Technology and Mechatronics Engineering Conference (ITOEC).

[11] Liu X., Hong Z., Shi W., Guo X. (2023). Image-processing-based subway tunnel crack detection system. Sensors.

[12] Hu D., Tian T., Yang H., Xu S. Wall Crack Detection Based on Image Processing. Proceedings of the 2012 Third International Conference on Intelligent Control and Information Processing (ICICIP).

[13] Jang K., Kim N., An Y.K. (2019). Deep Learning–Based Autonomous Concrete Crack Evaluation Through Hybrid Image Scanning. Struct. Health Monit..

[14] Nguyen A., Nguyen C.L., Gharehbaghi V., Perera R., Brown J., Yu Y., Tran M.T. (2022). A Computationally Efficient Crack Detection Approach Based on Deep Learning Assisted by Stockwell Transform and Linear Discriminant Analysis. Eng. Struct..

[15] Padsumbiya M., Brahmbhatt V. (2022). Automatic Crack Detection Using Morphological Filtering and CNNs. J. Soft Comput. Civ. Eng..

[16] Wu X., Liu Z., Huang M. A Tunnel Crack Extraction Method Based on Point Cloud. Proceedings of the 2022 3rd International Conference on Geology, Mapping and Remote Sensing (ICGMRS).

[17] Altabey W.A., Noori M., Wang T., Ghiasi R., Kuok S.-C., Wu Z. (2021). Deep Learning-Based Crack Identification for Steel Pipelines by Extracting Features from 3D Shadow Modeling. Appl. Sci..

[18] Tang J., Gu Y. Automatic Crack Detection and Segmentation Using a Hybrid Algorithm for Road Distress Analysis. Proceedings of the 2013 IEEE International Conference on Systems, Man, and Cybernetics, ICSMC.

[19] Xu H., Tian Y., Lin S., Wang S. Research of Image Segmentation Algorithm Applied to Concrete Bridge Cracks. Proceedings of the 2013 IEEE Third International Conference on Information Science and Technology (ICIST).

[20] Li B., Guo H., Wang Z., Li M. (2022). Automatic Crack Classification and Segmentation on Concrete Bridge Images Using Convolutional Neural Networks and Hybrid Image Processing. Intell. Transp. Infrastruct..

[21] Zhou Q., Qu Z., Li Y.X., Ju F.R. (2022). Tunnel Crack Detection with Linear Seam Based on Mixed Attention and Multiscale Feature Fusion. IEEE Trans. Intell. Transp. Syst..

[22] Yang X. Research on Bridge Crack Recognition Algorithm Based on Image Processing. Proceedings of the 2022 IEEE 5th International Conference on Knowledge Innovation and Invention (ICKII ).

[23] Sabouri M., Mohammadi M. (2023). Hybrid Method: Automatic Crack Detection of Asphalt Pavement Images Using Learning-Based and Density-Based Techniques. Int. J. Pavement Res. Technol..

[24] Li L.F., Wang N., Wu B., Zhang X. (2021). Segmentation Algorithm of Bridge Crack Image Based on Modified PSPNet. Laser Optoelectron. Prog..

[25] Fan Z., Li C., Chen Y., Wei J., Loprencipe G., Chen X. (2020). Automatic Crack Detection on Road Pavements Using Encoder-Decoder Architecture. Materials.

[26] Zhang H., Zhang A.A., Dong Z., He A., Liu Y., Zhan Y., Wang K.C.P. (2024). Robust Semantic Segmentation for Automatic Crack Detection Within Pavement Images Using Multi-Mixing of Global Context and Local Image Features. IEEE Trans. Intell. Transp. Syst..

[27] Belloni V., Sjölander A., Ravanelli R., Crespi M., Nascetti A. (2020). Tack Project: Tunnel and Bridge Automatic Crack Monitoring Using Deep Learning and Photogrammetry. Int. Arch. Photogramm. Remote Sens. Spat. Inf. Sci..

[28] Wu S., Xiong A., Luo X., Lai J. Bridge Crack Detection Based on Image Segmentation. Proceedings of the 2022 5th International Conference on Advanced Electronic Materials, Computers and Software Engineering (AEMCSE).

[29] Zhao F., Chao Y., Liu X., Li L. A Novel Crack Segmentation Method Based on Morphological-Processing Network. Proceedings of the 2022 15th International Congress on Image and Signal Processing, BioMedical Engineering and Informatics (CISP-BMEI).

[30] Man K., Liu R., Liu X., Song Z., Liu Z., Cao Z., Wu L. (2022). Water leakage and crack identification in tunnels based on transfer-learning and convolutional neural networks. Water.

[31] Su H., Wang X., Han T., Wang Z., Zhao Z., Zhang P. (2022). Bridge Crack Detection Using Full Attention U-Net. Buildings.

[32] Luan S., Gao X., Wang C., Zhang H. (2023). CrackF-Net: Pixel-Level Segmentation for Pavement Cracks. J. Electron. Imaging.

[33] Liang Z., Liu W., Yao R. (2016). Contrast Enhancement by Nonlinear Diffusion Filtering. IEEE Trans. Image Proc..

[34] Lin Y.H., Lu Y.C. (2022). Low-Light Enhancement Using a Plug-and-Play Retinex Model With Shrinkage Mapping for Illumination Estimation. IEEE Trans. Image Process..

[35] Yu S.Y., Zhu H. (2019). Low-Illumination Image Enhancement Algorithm Based on a Physical Lighting Model. IEEE Trans. Circuits Syst. Video Technol..

[36] Pu T., Zhu Q. (2024). Non-Uniform Illumination Image Enhancement via a Retinal Mechanism Inspired Decomposition. IEEE Trans. Consum. Electron..

[37] Wang S., Luo G. (2018). Naturalness Preserved Image Enhancement Using a Priori Multi-Layer Lightness Statistics. IEEE Trans. Image Process..

[38] Guo C., Li C., Guo J., Loy C.C., Hou J., Kwong S., Cong R. Zero-Reference Deep Curve Estimation for Low-Light Image Enhancement. Proceedings of the 2020 IEEE/CVF Conference on Computer Vision and Pattern Recognition (CVPR).

[39] Jiang Y., Gong X., Liu D., Cheng Y., Fang C., Shen X., Yang J., Zhou P., Wang Z. (2021). EnlightenGAN: Deep Light Enhancement without Paired Supervision. IEEE Trans. Image Process..

[40] Chen C., Chen Q., Xu J., Koltun V. Learning to See in the Dark. Proceedings of the 2018 IEEE/CVF Conference on Computer Vision and Pattern Recognition (CVPR).

[41] Ronneberger O., Fischer P., Brox T. U-Net: Convolutional Networks for Biomedical Image Segmentation. Proceedings of the 18th International Conference on Medical Image Computing and Computer-Assisted Intervention (MICCAI).

[42] Zunair H., Hamza A.B. Masked Supervised Learning for Semantic Segmentation. Proceedings of the 2022 British Machine Vision Conference (BMVC).

